# Supplementary material for: Characterizing two outbreak waves of COVID-19 in Spain using phenomenological epidemic modelling
Source: PLoS One. 2021 Jun 24;16(6):e0253004. doi: 10.1371/journal.pone.0253004 (PMC8224976; doi:10.1371/journal.pone.0253004)
Supplement: Attachment — Submitted filename: PONE-S-20-48031_response-to-reviewers_rev1.docx [file pone.0253004.s001.docx]

**Review Notes**

Dear Academic Editor of PLOS ONE,

First of all, I like to thank the reviewers for the received comments in order to improve the paper. I have included the corrections according to the reviewer’s comments as shown below, and I have tried to do my best.

In this revision note, reviewer’s comments are written in orange, and my responses are written in green. I also provide a marked-up copy of my manuscript that highlights the changes made to the original version

We hope that now the paper meets with the requirements to be published in Computer Networks Journal.

Yours sincerely,

Miguel Lopez

**Reviewer #1 comments**

Here particular aspects to improve the paper taking into account previous general comments:

There are few references and the introduction can be improved taking into account previous works.

- New references are added, and all of them are included in the introduction, mentioned as pervious works.

Line 20. How long? Relative to what?

- How long and relative to what is defined.

Line 73. Change the word current. If people look, the current situation in Spain would disagree with the result presented here and specially with the 2nd wave.

- The word *current* is supressed.

Line 87 fix the name

- The name was fixed.

Line 141 add reference and assumptions for this equation. This formula includes final epidemic size, which can't be obtained with the proposed models. Please explain this and the limitations.

- Reference and assumptions for this equation is added.

Line 213. Is a good comment.

- No changes were required.

Section 4.1. Explain why and how you chose the specifics early epidemic period? Notice that this would change the values of the estimated parameters.

- Why and how you chose the specifics early epidemic period is explained.

Section 4. For the first and second waves, the periods are different. Show in table Co for each wave.

- For the first and second waves, the different periods and Co are shown in the table.

Fig 6. The bootstrapping plots (parameter space) can show correlations between the parameters and asses if the parameters are identifiable. If this is not true, then there are infinite sets of parameter values such the fit is equally good. In this case, it seems that parameters are correlated, please show bootstrap plots in the parameter spaces.

- The Fig 6. Is divided in Fig 5. and Fig 10, increasing their size to depict the bootstrapping plots.

Divide the section in 1st wave and 2nd wave to see the differences more clearly.

- This section is divided in 1st wave and 2nd wave. This implies an increase in the number of the pages of the paper, as well as the change in the number of figures.

For each wave summarize initial condition, parameters, period, results in a Table.

- For the first and second waves, initial condition, parameters, period, results are shown in the table.

Line 377. I would not use final epidemic size here since as the authors mention this method is only valid for the early period. Moreover, as the authors mention this model does not take into account variation on social behaviors and NPI.

- A clarification and comment is included at this point.

Line 406. This paragraph needs to be changed carefully. The models presented here can't do long term forecast. This has been already mentioned by several studies and the media and people in general are blaming the models and forecasting. Just looking the current data of Spain it can be seen that extrapolation is wrong. However, still models are useful for other aspects. Please emphasize limitations of the study, and focus on what can be done with these limited models.

- This paragraph is revised and changed. Some clarification and comments are included at this point.

Lines 415. A deeper discussion would be useful regarding the unreported cases and the effect on the Ro and compare with other studies, Underreporting is a common issue an should be discussed. Authors should add some discussion of the asymptomatic cases and the findings n this study. Finally, discuss about the estimations given in the article and how this is affected by the infectious period, incubation period, proportion of asymptomatic, initial amount of recovered people

- This paragraph is changed. Some clarification and comments are included at this point.
